# Breast Milk Hormones and Regulation of Glucose Homeostasis

**DOI:** 10.1155/2011/803985

**Published:** 2011-05-05

**Authors:** Francesco Savino, Stefania Alfonsina Liguori, Miriam Sorrenti, Maria Francesca Fissore, Roberto Oggero

**Affiliations:** Department of Pediatrics, Regina Margherita Children's Hospital, University of Turin, 10126 Turin, Italy

## Abstract

Growing evidence suggests that a complex relationship exists between the central nervous system and peripheral organs involved in energy homeostasis. It consists in the balance between food intake and energy expenditure and includes the regulation of nutrient levels in storage organs, as well as in blood, in particular blood glucose. Therefore, food intake, energy expenditure, and glucose homeostasis are strictly connected to each other. Several hormones, such as leptin, adiponectin, resistin, and ghrelin, are involved in this complex regulation. These hormones play a role in the regulation of glucose metabolism and are involved in the development of obesity, diabetes, and metabolic syndrome. Recently, their presence in breast milk has been detected, suggesting that they may be involved in the regulation of growth in early infancy and could influence the programming of energy balance later in life. This paper focuses on hormones present in breast milk and their role in glucose homeostasis.

## 1. Introduction

It is well known from researches and literature data that food intake, energy expenditure, and glucose homeostasis are regulated by hypothalamic areas in which a variety of peripheral signals converge [1]. In conditions of excessive energy storage or nutrient abundance, specific hypothalamic areas reduce food intake, increase energy expenditure, and diminish endogenous glucose production. Therefore, in response to a reduction in energy stores or circulating nutrients, the brain promotes a positive energy balance to restore and maintain energy and glucose homeostasis. 

Insulin and leptin, which act as adiposity signals, play a pivotal role in the central regulation of energy homeostasis [2]. Both hormones circulate at levels proportional to body fat and regulate food intake and energy expenditure by interacting with their respective receptors [3]. They also influence glucose metabolism, acting at a peripheral and central level [4]. It is now recognized that, as well as leptin, adipose tissue produces other bioactive peptides called adipokines, which both influence autocrine and paracrine function of adipocytes and exert an endocrine function by their release in bloodstream [5]. Adiponectin has been hypothesized to act as a link between accumulated fat mass and altered insulin sensitivity even though the contribution to the development of insulin resistance is complex and not fully understood. Particularly, it has been observed that adiponectin increases fatty acid (FA) oxidation and decreases triglyceride storage in skeletal muscle, which may explain, in part, the insulin-sensitizing effect of this cytokine [6]. Conversely, resistin has been implicated in impairing insulin sensitivity in rodents [7], while its role in the development of insulin resistance in humans is still controversial [8]. The mechanism involved seems to be the impairing of glucose uptake in skeletal muscle, while there is little information regarding the effect of resistin on skeletal muscle FA metabolism [9]. As basal levels of insulin and leptin are widely accepted to be adiposity signals, also ghrelin has been hypothesized to have the same role. An association between basal ghrelin levels and levels of adiposity has been postulated, and the presence of receptors of this hormone regulating energy homeostasis in neural circuits has been demonstrated [1]. Also ghrelin is involved in glucose metabolism, and there is a relationship between ghrelin and insulin levels.

Early nutrition could exert both short- and long-term effects on the programming of metabolic development and growth. In this contest, it is interesting to know that breast milk (BM) contains hormones such as leptin, adiponectin, resistin, and ghrelin, which play a role in energy balance regulation and glucose homeostasis. These hormones may be involved in the regulation of growth and development in the neonatal age and infancy and could influence the programming of energy balance regulation in childhood and adulthood.

Further diet-related differences during infancy in serum levels of factors involved in energy metabolism might explain anthropometric differences and differences in dietary habits between breast-fed (BF) and formula-fed (FF) infants also later in life and may thus have long-term health consequences. In this context, the recent finding of higher leptin levels and lower ghrelin levels in BF infants than in FF ones suggests that differences in hormonal values together with different protein intake could account for the differences in growth between BF and FF infants both during infancy and later in life [10].

Here, we review data related to hormones contained in mother's milk and their role in the regulation of glucose homeostasis (Table 1).

## 2. Leptin

Leptin, the product of the obesity (*ob*) gene, is a 16 kDa peptide that is primarily produced by white adipose tissue along with other adipokines [11]. Extra-adipose leptin synthesizing sites are hypothalamus, pituitary, skeletal muscle, stomach, liver, and placenta [12]. Moreover, leptin is present in human milk, produced and secreted by mammary epithelial cells in milk fat globules [13]. Recent data about leptin concentration in BM showed variations, mainly according to the BM fractions and the sample treatments used, with mean values ranging from 0.2 to 73.22 ng/mL [14].

Circulating leptin concentration has been reported to correlate closely with body mass index (BMI) and the total amount of body fat [15]. Leptin is secreted in a pulsatile manner and has significant plasma diurnal variation, with higher levels in the evening and early morning hours [16]. 

The normal range for plasma leptin in healthy adult subjects is 3–5 ng/mL, while concentrations reported in obese humans are much higher, in the range of 8–90 ng/mL [17]. In a study conducted on healthy infants, mean serum leptin concentration was 3.35 ng/mL [10].

Leptin's main function consists in signalling to the brain how much fat is being stored and to regulate food intake. In the arcuate nucleus of hypothalamus, leptin stimulates the secretion of anorexigenic peptides, such as proopiomelanocortin (POMC) and cocaine- and amphetamine-regulated transcript (CART), and inhibits the secretion of orexigenic peptides, such as neuropeptide Y (NPY) and the agouti gene-related protein (AgRP) [18]. In addition to its central effects, leptin interacts with peripheral tissues including skeletal muscle, liver, and pancreas, modulating glucose and fat metabolism [6]. Leptin mediates its effects by binding to specific receptors (ObRs) expressed in the brain and in peripheral tissues. The binding of leptin to its receptor activates several signal transduction pathways, including Janus kinase signal transducer and activator of transcription 3 (JAK-STAT3), which is important for the regulation of energy homeostasis [19], and phosphatidylinositol 3-kinase (PI3K), which is important for the regulation of both food intake and glucose homeostasis [20]. In fact, recent evidence underlines that, besides its peripheral action, leptin plays also an important role in the hypothalamic control of glucose metabolism. For this action the activation of the insulin receptor substrate-2 (IRS-2) and hypothalamic PI3K pathway, which is the major intracellular mediator of insulin action, is required [21]. Therefore, the arcuate nucleus of hypothalamus is an important site mediating the effect of leptin in the control of glucose metabolism, for its neuronal subsets that can control food intake, energy expenditure, and insulin sensitivity. At this level, leptin and insulin inhibit NPY/AgRP neurons and activate POMC ones. Both neuronal subsets project to paraventricular nucleus (PVN) and lateral hypothalamic area (LHA) and from these to other hindbrain areas such as nucleus of the solitary tract (NTS), generating a vagal signal to the liver to regulate hepatic glucose production [1]. 

Leptin may also increase energy expenditure, rising sympathetic nerve activity and activating brown adipose tissue thermogenesis; however, these effects have been confirmed only in mice [22].

### 2.1. Role of Leptin on Glucose Homeostasis and Insulin Action

Studies in animals and humans show that leptin plays a pivotal role in the regulation of glucose homeostasis. In lean rats, systemic or central leptin infusion leads to a rapid redistribution of hepatic glucose fluxes with a stimulation of gluconeogenesis and inhibition of glycogenolysis [23]. Experimental studies in rats show that leptin can reduce insulin secretion [24, 25]. Also in studies on human pancreatic beta cells, an inhibitory effect of leptin on insulin secretion was detected, while insulin promotes leptin secretion [26].

Leptin improves insulin sensitivity reducing intracellular lipid levels in skeletal muscle, liver, and pancreatic beta cells. In muscle, insulin sensitization is achieved through malonyl CoA inhibition, which increases transport of  FA into mitochondria for beta oxidation. These changes are partially mediated by central sympathetic activation of adrenergic receptors [27].

States of congenital leptin deficiency because of mutations of the leptin gene have been associated with severe obesity, glucose intolerance, diabetes, and insulin resistance in humans. Leptin treatment in humans with congenital leptin deficiency has been shown to improve glycemic control and hyperinsulinemia [28]. Similarly, lipodystrophic syndromes, with complete or partial absence of subcutaneous adipose tissue, have been associated with low leptin concentrations and diabetes. Leptin administration in replacement doses in HIV-positive patients with partial lipoatrophy improved glycemia, dyslipidemia, and insulin resistance [29].

Oral administration of this hormone at doses close to the physiological concentration in milk reduced food intake in newborn rats and was absorbed by the rat's stomach during neonatal development thereby triggering downregulation of endogenous leptin production [30]. In a study of rat pups monitored into adulthood, animals given physiological doses of oral leptin during lactation had lower body weight and fat content than untreated animals, which suggests that leptin could have long-term effects on body weight regulation [31]. More recently, leptin-treated animals were found to have lower body weight in adulthood, to eat fewer calories, to have higher insulin sensitivity, and to show a lower preference for fat-rich food than their controls [32]. Farooqi et al. observed that administration of exogenous leptin to leptin-deficient children resulted in a remarkable decrease in their energy intake and a dramatic loss of fat mass while maintaining lean body mass [33]. Furthermore, it has been observed that the administration of exogenous leptin fails to reduce adiposity significantly in most cases of human obesity that are characterized by increased adipocyte leptin content and high circulating leptin levels, reflecting a state of leptin resistance [34].

The concept of “leptin resistance” was introduced when increased adipose leptin production was observed in obese individuals, who were not leptin deficient; the failure of high leptin levels to suppress feeding and decrease body weight or adiposity to prevent or mitigate obesity suggests a relative resistance to the catabolic effects of leptin action in obesity [35]. Therefore, obese subjects show high leptin levels, while congenital leptin deficiency is associated with obesity (Table 2).

### 2.2. Leptin and Glucose Homeostasis in Newborns and Infants

Many experimental data suggest that leptin role in the regulation of food intake and energy homeostasis begins in early life, when this hormone also controls fetal growth and development [36]. It has been also observed that leptin is an essential factor for the development of hypothalamic pathways involved in the regulation of energy balance and that this activity is restricted to a neonatal critical period. Leptin is an intrinsic part of the maturation process of pathways involved in appetite regulation; in mice, there is a surge in leptin soon after birth where the timing is critical for the correct development of the pathways [37]. 

At birth, serum leptin concentrations seem to be related to the amount of body adipose tissue. Positive correlations between cord blood leptin levels, birth weight, BMI, insulin and insulin-glucose ratio have been reported for full-term infants and preterm ones [38]. In appropriate-for-gestational age (AGA) term infants, mean concentration of leptin was 4.01 ± 3.5 ng/mL, lower than that in large-for-gestational age (LGA) term infants in which cord leptin concentration was 7.3 ± 3.8 ng/mL. Preterm infants had a mean leptin level of 1.8 ± 0.97 ng/mL, and the concentration observed in IUGR infants was 6.5 ± 3.9 ng/mL [39].

Cord blood leptin concentration has been observed to be related to rates of intrauterine growth, suggesting a possible role of leptin in promoting fetus growth: small-for-gestational age (SGA) neonates have lower leptin levels at birth (3.3 ± 0.5 *μ*g/L) than AGA infants (14.5 ± 2.8 *μ*g/L), and LGA neonates have higher leptin levels than other infants (35.7 ± 8.0 *μ*g/L) [40]. 

A trend for higher leptin levels in LGA infants of diabetic mothers than in LGA ones of nondiabetic mothers was observed. Both groups of LGA infants showed lower glucose levels [41].

Cord blood leptin seems to be a predictor of weight gain also in later life; in fact, lower cord blood leptin levels have been observed to be associated with smaller size at birth but more pronounced weight gain in the first 6 months of life and higher BMI at 3 years of age [42].

As concerns infants, higher serum leptin concentrations were detected during the first months of life in breast-fed infants (5.1 ± 3.6 ng/mL) compared to those who were fed with formula (4.2 ± 4.9 ng/mL) [43].

## 3. Adiponectin

Adiponectin, discovered in 1995, is the most abundant adipose-specific protein, and its multiple functions have started to emerge in recent years [44]. In adults, plasma adiponectin concentration ranges from 0.5 to 30 *μ*g/mL, 1000-fold higher than the concentrations of other hormones, such as insulin or leptin. 

A reduction in adiponectin expression has been associated with insulin resistance, whereas administration of adiponectin is accompanied by an increase in insulin sensitivity. Adiponectin production is regulated by the peroxisomal proliferator-activated receptor-*γ* (PPAR-*γ*), a nuclear receptor whose expression in liver and muscle seems to be important in the mediation of obesity-related insulin resistance [45]. Adiponectin binds to two different receptors: AdipoR1 is abundantly expressed in skeletal muscle, while AdipoR2 is predominantly expressed in the liver. It has been observed that suppression of both AdipoR1 and AdipoR2 expression abolishes adiponectin binding and decreases PPAR*α* ligand activity, fatty acid oxidation, and glucose uptake [46].

Adiponectin exists in plasma in oligomeric complexes, consisting of trimers (low molecular weight), hexamers (medium molecular weight), and large multimers of 12–18 subunits (high molecular weight (HMW)) [47]. HMW adiponectin mediates insulin sensitization in peripheral tissues and seems to be the most biologically active form of adiponectin in terms of glucose homeostasis [48]. HMW adiponectin seems to be a better predictor of insulin resistance than total adiponectin [49]; in particular, the ratio of HMW to total adiponectin or HMW to LMW may provide a better reflection of peripheral insulin sensitivity than total adiponectin alone [50]. Compared with total adiponectin, HMW adiponectin is recognized as a predictor of risk of developing type 2 diabetes and even a stronger predictor than total adiponectin [51]. In particular, high HMW adiponectin concentrations are strongly associated with lower risk of type 2 diabetes [52].

Adiponectin is present in BM, with an average concentration of approximately 19 ng/mL (range, 4 to 88 ng/mL) [53]. Martin et al. reported that immunoreactive adiponectin levels in human milk were significantly higher than leptin levels, and that they decreased with the duration of lactation [54]. Bronský et al. looked for adipose-tissue-generated proteins that are related to lipid metabolism in BM and found adiponectin, adipocyte fatty acid-binding protein, and epidermal fatty acid-binding protein [55].

Interestingly, adiponectin was found to be more abundant in cord blood (30.6 mg/L) than in either BM (10.9 *μ*g/L) or maternal serum (8.6 mg/L) [56], confirming data from previous observational studies showing that cord blood adiponectin levels are higher than those seen in adults [57]. Similar cord blood adiponectin levels (28.5 *μ*g/mL) were detected in a recent study, in which it was found to be directly associated with birth weight for gestational age, inversely associated with weight gain in the first 6 months of life, and to predict an increase in central adiposity at the age of 3 years [58].

Given the biological properties of adiponectin, its presence in BM and the expression of adiponectin receptor 1 in the small intestine of neonatal mice [59], not only adipose-tissue-produced adiponectin but also milk adiponectin, may affect infant growth and development. 

### 3.1. Role of Adiponectin on Glucose Homeostasis and Insulin Action

Adiponectin is involved in the regulation of glucose metabolism. In the liver, adiponectin increases insulin action by suppressing FA influx and increasing fat oxidation, decreasing gluconeogenesis, and reducing ectopic fat deposition in the liver [60]. In muscle cells, adiponectin increases glucose utilization by increasing FA oxidation and thus decreasing the triglyceride content in skeletal muscle [61]. Adiponectin-linked insulin sensitization is mediated, at least in part, by activation of adenosine monophosphate-activated protein kinase (AMPK) in skeletal muscles and the liver, which increases FA oxidation and reduces hepatic glucose production [62].

Low adiponectin seems to be a cause rather than a consequence of hyperinsulinaemia. Prospective studies show that low levels of adiponectin precede a decrease in insulin sensitivity [63]. Insulin may suppress adiponectin levels [64]. 

Serum adiponectin is decreased in insulin resistance and type 2 diabetes, independently of adiposity [65]. Genetic polymorphisms responsible for hypoadiponectinaemia are associated with insulin resistance [66]. Plasma adiponectin levels are inversely related to the degree of adiposity; in fact, obese individuals have decreased plasma adiponectin concentrations, and females, who are proportionately fatter than males, show lower adiponectin levels [67] (Table 2).

### 3.2. Adiponectin and Glucose Homeostasis in Newborns and Infants

Circulating adiponectin levels correlate negatively with the degree of adiposity in children aged between 5 and 10 years [68]. In contrast, in full-term neonates, during the first few days of life, serum and plasma adiponectin levels correlate positively with birth weight and length, neonatal adiposity, and circulating levels of leptin [69]. Furthermore, adiponectin concentrations in cord blood are higher than those reported in adolescents and in adults [70]. In preterm infants, serum adiponectin levels are lower than those in full-term infants, correlate positively with body weight, and increase with postnatal age, which suggests a metabolic adaptation to premature extrauterine life [71]. Blood adiponectin levels increase with postnatal age in premature infants, suggesting a rapid, as yet unexplained, metabolic adaptation to premature extrauterine life [72]. 

In prepubertal children who were born SGA, serum adiponectin levels were comparable to AGA children [73]. In another study, the SGA < 3rd percentile children had higher adiponectin than AGA children [74].

## 4. Resistin

The peptide hormone resistin, also called FIZZ3, is an adipocyte-derived secretory factor which was first identified as a novel transcript produced exclusively by adipocytes and has been shown to play a significant role in obesity-induced insulin resistance [7]. The name was considered appropriate because resistin was reported to antagonize insulin action in cells *in vitro* as well *in vivo*. Resistin is expressed within adipocytes of rodents [7] and mainly in macrophages of humans [75], and its production is increased with feeding and obesity and decreased by PPAR*γ* ligands [76]. It is also found in serum in mice and in humans [7, 77]. 

Resistin gene expression has been demonstrated in human placental tissues, mainly in trophoblastic cells, in particular in term placental tissue, it is more prominent than in the first trimester chorionic tissue [78]. Ilcol et al. investigated if human milk was a possible source of resistin. They showed that resistin is present in human BM, and its concentration in BM decreases with time during lactation, with levels ranging from 1710 ± 68 pg/mL at 1–3 postpartum days to 670 ± 18 pg/mL at 91–180 postpartum days [79]. 

Expression of resistin is low during food deprivation and increases greatly during refeeding. Resistin plays a role as a regulator of glucose homeostasis, inhibits adipocyte differentiation, and may function as a feedback regulator of adipogenesis [80].

### 4.1. Role of Resistin on Glucose Homeostasis and Insulin Action

Steppan et al. suggested that resistin is a unique hormone whose effects on glucose metabolism are antagonistic to those of insulin [7]. Two independent studies, in which recombinant resistin was administered to rodents, argue that resistin can increase blood glucose levels and cause insulin resistance. Indeed, Rajala et al. showed that in rodents acute resistin increased glucose production, possibly by activation of the glucose-6-phosphatase gene and without apparent changes in glucose utilization by skeletal muscle and adipose tissue [81]. Administration of resistin to mice resulted in increased glucose production and blood glucose levels and impaired insulin action; neutralization of resistin by injection of antibodies in diet-induced obese mice decreased blood glucose levels and improved insulin sensitivity [7].

In rat skeletal muscle cells, it has been shown that resistin does not alter insulin receptor signaling but affects insulin-stimulated glucose uptake, presumably by decreasing the intrinsic activity of cell surface glucose transporters [82]. In mature 3T3-L1 adipocytes, resistin reduces insulin-stimulated glucose uptake by activating SOCS3, which is an inhibitor of insulin signaling [83]. These studies suggest that resistin may contribute to insulin resistance, and its effects are being mediated at target tissue such as liver, skeletal muscle, and adipose tissue. Interestingly, it has been observed that insulin seems to inhibit resistin secretion, while resistin induces insulin resistance, in an insulin-resistin-insulin sensitivity positive feedback loop [84]. 

The long-term *in vivo* function of resistin was studied in mice knockout for resistin gene. In resistin-deficient (R^−/−^) mice neither R-mRNA nor resistin protein were expressed in white adipose tissue, and resistin was not found in R^−/−^ serum. Blood glucose levels in the R^−/−^ mice, when fed a high-fat diet, were 20% to 30% lower than in wild-type (WT) controls, and glucose production was reduced compared with WT control [85].

Regulation of resistin expression has been studied in response to insulin and glucose. Experimental studies showed that resistin mRNA levels are increased in response to acute hyperglycaemia and decrease in response to hyperinsulinaemia in mice [86] and in 3T3-L1 adipocytes [87].

### 4.2. Resistin in Obesity and Diabetes

Resistin expression has been analysed in rodent models of obesity and diabetes, suggesting that it is not clear if serum resistin levels in obesity genetic models (ob/ob and db/db) as well as in a diet-induced model of diabetes and obesity are elevated or decreased [7, 86]. This finding suggests that resistin may have little or no contribution to insulin resistance and that there is already conflicting evidence in relation to changes in resistin expression in dietary and genetic models of rodent obesity.

Owecki et al. examined correlations between serum resistin concentrations and the degree of human obesity and insulin sensitivity. Obese subjects showed higher resistin concentrations than nonobese controls, but despite these different concentrations, no relationship between resistin concentration and insulin resistance has been found [88]. In obese children serum resistin levels did not differ from that of control subjects [89]. 

In a study conducted on patients with type 2 diabetes, serum resistin concentration was significantly higher than in normal subjects [90] (Table 2).

### 4.3. Resistin and Glucose Homeostasis in Newborns and Infants

Cord blood levels of resistin and their postnatal changes in term AGA neonates have been investigated, showing high resistin levels at birth (8.63 ± 2.94 ng/mL), similar to those on the 4th day of life (7.87 ± 4.02 ng/mL). These findings suggest that this hormone may play a role in maintenance of metabolic neonatal homeostasis [91]. In a sample of 37 neonates born at term mean, umbilical serum resistin concentration was 21.34 ± 1.07 ng/mL, ranging from 10.61 to 40.81 ng/mL [92]. Moreover, plasma resistin concentrations were significantly higher in term (12.1; range: 7.8–17.7 ng/mL) than in preterm infants (5.2; range: 3.4–12.7 ng/mL), suggesting that high circulating resistin levels at term gestation could be advantageous to the infant by promoting hepatic glucose production and preventing hypoglycemia after birth [93]. In term newborns of mothers with insulin-dependent diabetes, serum resistin is significantly suppressed, suggesting that the metabolic hormonal system is probably operational in fetal and early postnatal life. The suppressive effect of insulin on resistin may partially explain the excess accumulation of adipose tissue in infants of diabetic mothers by reducing the inhibitory effect of resistin on adipogenesis [94].

## 5. Ghrelin

Ghrelin was identified as an endogenous ligand for the growth hormone secretagogue receptor type 1a (GHS-R 1a) [95]. It is a peptide hormone of 28 amino acids with an octanoyl group on the serine in position 3, which is crucial for its biological activity [96]. GHS-R 1a is expressed mainly in the pituitary and hypothalamus. Ghrelin is produced predominantly by the stomach, by the X/A-like endocrine cells, and by many other tissues such as pituitary, hypothalamus, bowel, lung, heart, pancreas, kidney, placenta, and testis [97]. As suggested by its widespread distribution, ghrelin has a wide spectrum of biological activities beyond the stimulation of growth hormone release [98, 99]. Indeed, ghrelin regulates gastric motility, acid secretion, pancreatic secretion, glucose and lipid metabolism, and cell proliferation and exerts cardiovascular and anti-inflammatory effects [100, 101]. Ghrelin has a short-term action on energy homeostasis, stimulating appetite and food intake, and a long-term effect, enhancing fat-mass deposition and body-weight gain [102]. Ghrelin secretion is increased by fasting and in response to weight loss, while it decreases under positive energy-balance conditions, such as food intake and obesity [103]. 

Ghrelin is present in both term and preterm human BM; its levels are higher in whole milk than in skim milk, with median ghrelin level in whole BM of 2125 pg/mL [104]. Aydin et al. showed lower concentrations in colostrum (70.3 ± 18 pg/mL), transitional milk (83.8 ± 18 pg/mL), and mature milk (97.3 ± 13 pg/mL) [105]. Acylated ghrelin has also been reported in BM; its concentrations increase during lactation and are significantly related to serum ghrelin concentrations in BF infants; active and total ghrelin concentrations in BM were lowest (450 ± 25 and 880 ± 80 pg/mL, resp.) at 0–3 days, whereas they increased progressively during 180 days of lactation period to 801 ± 43 and 3250 ± 380 pg/mL at 91–180 days postpartum [106].

### 5.1. Role of Ghrelin on Glucose Homeostasis and Insulin Action

There are contrasting reports concerning the influence of ghrelin on insulin secretion. Earlier experiments observed that ghrelin stimulates the release of insulin in isolated rat pancreatic islets [107]. Subsequently, ghrelin has been shown to inhibit insulin secretion in rat pancreatic *β* cells *in vitro *[108], and this effect has been confirmed also in studies *in vivo* in several species [109]. In agreement with the assumption that ghrelin negatively modulates pancreatic *β*-cell secretion, recently it has been demonstrated that in humans ghrelin induces a significant increase in plasma glucose levels that is followed, surprisingly, by a reduction in insulin secretion [110]. Studies *in vitro* have shown that ghrelin inhibits insulin effects, inhibiting glycogen synthesis and promoting gluconeogenesis [111]. Studies in human subjects found that administration of ghrelin induces hyperglycaemia and reduces insulin secretion, probably through a direct glycogenolytic effect [112]. Also, ghrelin secretion may be suppressed, at least in part, by an increased plasma glucose level as well as by insulin, as shown by hyperinsulinemic euglycemic clamp studies in healthy subjects [113]. These findings indicate that insulin is a physiological and dynamic modulator of plasma ghrelin and that insulinemia possibly mediates the effect of nutritional status on its concentration. 

Changes in plasma ghrelin concentrations have been reported in animal models of diabetes and in human diabetes type 1 and type 2. It has been shown in a model of streptozotocin-induced diabetes that plasma ghrelin concentration and preproghrelin mRNA expression in the stomach of the diabetic rats increased significantly whereas their gastric ghrelin level and the number of ghrelin-immunoreactive cells in the gastric fundus decreased significantly [114]. In type 1 diabetic patients decreased plasma ghrelin levels have been documented [115–117], and after prolonged insulin treatment or food intake, a suppression of plasma ghrelin in these patients has been shown [118, 119]. Also in children with insulin-treated type 1 diabetes, the plasma ghrelin levels were found to be lower than those measured in controls with a negative association of their plasma ghrelin levels with daily insulin dosage [116]. Further low levels of plasma ghrelin persisted during long-term insulin treatment [120]. 

The relationship between plasma ghrelin concentration and serum insulin level or insulin resistance in patients with type 2 diabetes is still controversial, even though a large number of studies indicated decreased plasma ghrelin levels in obese type 2 diabetic patients [121–124]. Moreover, a negative correlation between fasting plasma ghrelin and insulin was observed, suggesting that hyperinsulinaemia associated with insulin resistance may be an important determinant of decreased plasma ghrelin levels in patients with type 2 diabetes [125].

Although decreased plasma ghrelin levels may contribute to either the development of hyperinsulinaemia or to the restraint of weight gain in patients with type 2 diabetes, the lack of data about the cause-and-effect relationship makes it difficult to propose an exact role of ghrelin in the development of type 2 diabetes. It seems possible that decreased plasma ghrelin concentrations in type 2 diabetic patients may be due to a compensatory mechanism against diminished insulin action or against hyperglycaemia [126] (Table 2).

### 5.2. Ghrelin and Glucose Homeostasis in Newborns and Infants

Ghrelin has been detected in cord plasma as early as 30 weeks of gestation, suggesting a possible role in fetus developing [127]. 

In newborns, cord blood ghrelin levels are higher in preterm SGA babies (602 ng/L; 95% CI: 330–1211) than in preterm AGA ones (200 ng/L; 95% CI:164–245). Reduced ghrelin suppression and higher postprandial ghrelin levels in SGA infants could result in a sustained orexigenic drive and could contribute to postprandial catchup growth in these infants [128]. 

Also preterm newborns SGA show higher cord blood ghrelin levels than AGA ones [129], and newborns who had intrauterine growth restriction show low cord blood glucose concentrations and increased ghrelin concentrations [130]. 

It has been observed that the rate of weight gain over the first year in SGA infants correlate with a variable suppression of circulating ghrelin levels. In fact, SGA infants who showed poor catchup growth showed a larger decline in ghrelin concentrations measured during fasting and 10 minutes after intravenous (iv) glucose. Higher ghrelin levels or lower reductions in circulating levels following iv glucose were seen in SGA infants who showed greater infancy weight gain, suggesting that sustained orexigenic drive could contribute to postnatal catchup growth [131].

Studies on serum ghrelin levels in infants are scanty. In a study conducted on infants in the first 4 months of life, FF infants showed higher serum ghrelin levels compared to BF ones [132]. In a cohort of infants aged until 18 months, serum ghrelin correlated positively with fasting time, and a negative influence of insulin on ghrelin levels was observed [133]. A positive correlation between circulating ghrelin levels and fasting time was observed also in exclusively FF infants in the first 6 months of life [134].

## 6. Conclusions

Consistent evidence suggests that the complex mechanisms implicated in energy homeostasis involve the central nervous system and peripheral organs, connecting each other food intake, energy expenditure, and glucose homeostasis. Moreover, it seems that early-life nutrition and growth could program the set point of energy homeostasis later in life. Several hormones, such as leptin, adiponectin, resistin and ghrelin are involved in this complex regulation, and their presence in BM has been demonstrated, representing the link between breastfeeding and protection against metabolic-related disorders in later life. In fact, these hormones may be involved in the regulation of growth and development in the neonatal age and in infancy and could take part in the programming of energy balance in childhood and adulthood, and they may be implicated in the development of obesity, diabetes, and metabolic syndrome. 

Considering leptin, studies in animals and humans show that it plays a pivotal role in the regulation of glucose homeostasis. Leptin is implicated in the regulation of food intake and energy homeostasis in early life, when this hormone also controls fetal growth and development; particularly in neonatal period, it is also an essential factor for the development of hypothalamic pathways involved in the regulation of energy balance. 

Also adiponectin is involved in the regulation of glucose metabolism, increasing insulin action in liver and rising glucose utilization in skeletal muscle. This hormone plays a role since early life, and its serum levels correlate with neonatal growth parameters, adiposity, and circulating levels of leptin. 

As concerns resistin, it is a unique hormone whose effects on glucose metabolism are antagonistic to those of insulin. Particularly, insulin seems to inhibit resistin secretion, while resistin induces insulin resistance, in an insulin-resistin-insulin sensitivity positive feedback loop. This hormone may play a role in maintenance of metabolic neonatal homeostasis; high circulating resistin levels at term gestation could be advantageous to the infant by promoting hepatic glucose production and preventing hypoglycemia after birth.

Ghrelin induces a significant increase in plasma glucose levels that is followed, surprisingly, by a reduction in insulin secretion. Also ghrelin has possible role in early life growth and development, as it has been detected in cord plasma since 30 weeks of gestation, and its serum levels correlate with growth parameters in neonates and infants, sustaining an orexigenic drive and contributing to catchup growth. 

Data on bioavailability and metabolic effects of oral administration of these hormones are scanty as compared to hormones previously discovered such as insulin, GH, or thyroid hormone; in particular, effects of leptin or ghrelin administration are documented as reported above, while data about the other hormones are still limited.

Longitudinal investigations will shed light on the new hormones discovered in mother's milk and their potential protective effect on subsequent obesity and metabolic-related disorders, in particular in the control of glucose homeostasis.

## Figures and Tables

**Table 1:** Breast milk (BM) hormones and their action on glucose homeostasis.

	BM levels (range or mean value)	Blood glucose	Insulin secretion	Insulin action
Leptin	0.2–73.22 ng/mL	Reduces	Inhibits	Increases
Adiponectin	4–88 ng/mL	Reduces	Inhibits	Increases
Resistin	1745 pg/mL	Increases	?	Reduces
Ghrelin	97.3–3250 pg/mL	Increases	Inhibits	Reduces

**Table 2:** Hormonal modifications in type 2 diabetes and obesity.

	Type 2 diabetes	Obesity
Leptin	Decreased	Elevated
		Decreased (congenital leptin deficiency)
Adiponectin	Decreased	Decreased
Resistin	Elevated	Elevated
Ghrelin	Decreased	Decreased
